# Patient Characteristics and Telemedicine Use in the US, 2022

**DOI:** 10.1001/jamanetworkopen.2024.3354

**Published:** 2024-03-22

**Authors:** Eva Chang, Robert B. Penfold, Nancy D. Berkman

**Affiliations:** 1 Advocate Aurora Research Institute, Advocate Health, Milwaukee, Wisconsin; 2 Kaiser Permanente Washington Health Research Institute, Seattle; 3 RTI International, Research Triangle Park, North Carolina

## Abstract

**Importance:**

Telemedicine use was common during the COVID-19 pandemic, expanding many patients’ approaches to accessing health care. Of concern is whether telemedicine access was poorer among higher-needs and disadvantaged populations.

**Objective:**

To assess patient characteristics associated with telemedicine use and telemedicine mode and describe telemedicine visit experiences by telemedicine mode.

**Design, Setting, and Participants:**

This cross-sectional study included data from the 2022 Health Information National Trends Survey and included US adults with a health care visit. Data were analyzed from May to September 2023.

**Exposure:**

Patient characteristics.

**Main Outcomes and Measures:**

Any telemedicine visits vs in-person visits only; telemedicine mode (video vs audio-only). Multivariable logistic models assessed patient characteristics associated with telemedicine visits and mode. Bivariate analyses compared telemedicine experiences by mode.

**Results:**

The study included 5437 adult patients (mean [SE] age, 49.4 [0.23] years; 3136 females [53.4%]; 1928 males [46.6%]). In 2022, 2384 patients (43%) had a telemedicine visit; 1565 (70%) had a video visit while 819 (30%) had an audio-only visit. In multivariable models, older age (≥75 years: adjusted odds ratio [aOR], 0.63; 95% CI, 0.42-0.94), no internet use (aOR, 0.62; 95% CI, 0.48-0.81), and living in the Midwest (aOR, 0.50; 95% CI, 0.35-0.70) were negatively associated with having telemedicine visits. Female sex (aOR, 1.43; 95% CI, 1.12-1.83), having chronic conditions (aOR, 2.13; 95% CI, 1.66-2.73), and multiple health care visits (2-4 visits: aOR, 1.77; 95% CI, 1.23-2.54; ≥5 visits: aOR, 3.29; 95% CI, 2.20-4.92) were positively associated. Among individuals who used telemedicine, older age (65-74 years: aOR, 2.13; 95% CI, 1.09-4.14; ≥75 years: aOR, 3.58; 95% CI, 1.60-8.00), no health insurance (aOR, 2.84; 95% CI, 1.42-5.67), and no internet use (aOR, 2.11; 95% CI, 1.18-3.78) were positively associated with having audio-only visits. We observed no significant differences in telemedicine use or mode by education, race and ethnicity, or income. Patients’ experiences using telemedicine were generally similar for video and audio-only except more individuals who used audio-only had privacy concerns (20% vs 12%, *P* = .02).

**Conclusions and Relevance:**

In this cross-sectional study of adults with health care visits, many patients, including those with the greatest care needs, chose telemedicine even after in-person visits were available. These findings support continuing this care delivery approach as an option valued by patients. Differences were not observed by most common measures of socioeconomic status. Continued monitoring of telemedicine use is needed to ensure equitable access to health care innovations.

## Key Points

**Question:**

Which patients used telemedicine after in-person visits became more available post–COVID-19 restrictions?

**Findings:**

In this cross-sectional study of 5437 US adults with health care visits in 2022, 43% used telemedicine (70% video visits; 30% audio-only visits). Patients who used telemedicine were more likely to have more health care needs while video telemedicine visits were less likely among older patients and those without internet; no differences were observed by education, race and ethnicity, or income.

**Meaning:**

These findings suggest that while many patients chose to use telemedicine when in-person visits are available, telemedicine access, particularly video visits, was less likely among key populations who experience barriers to accessing care.

## Teaser

This cross-sectional study assesses patient characteristics associated with telemedicine use and telemedicine mode and describes telemedicine visit experiences by telemedicine mode.

## Introduction

During the COVID-19 pandemic, telemedicine emerged as an important approach for delivering health care. Telemedicine use increased rapidly after COVID-19 was declared a public health emergency.^1^ Overall outpatient service rates were able to rebound quickly, largely due to the shift from in-person to telemedicine visits; approximately 50% of visits were through telemedicine during the pandemic’s peak in April 2020.^1,2,3^ While telemedicine visits have since declined, recent estimates suggest steady use. In 2021 and 2022, 20% to 39% of adults had telemedicine health care encounters.^4,5^

Several studies have shown patient willingness and desire to use video telemedicine; between 36% to 67% of patients would like to seek care using telemedicine in the future.^6,7,8,9^ Despite patients’ continued interest in telemedicine, equitable access remains a concern.^9,10,11,12,13,14,15,16^ Telemedicine offers the promise of improving access by removing financial and logistical barriers associated with transportation, work coverage, and childcare.^15^ However, limited access to the technology (particularly for video visits) and other internet-based services risks widening gaps in health care access rather than narrowing them. Several studies from early in the COVID-19 pandemic found less use of telemedicine visits and in fewer video telemedicine visits (than audio-only visits) among patients who were low-income, non-English–speaking, and older age.^12,13,16,17,18,19,20^ To our knowledge, few studies have examined how telemedicine use, and its modalities, has evolved.^5^

If telemedicine is to remain a common and important approach to health care delivery, it is necessary to understand who is using the technology successfully and why, and, conversely, uncover barriers in access. Using a nationally representative survey, our objective was to assess telemedicine use and modality among adults who had a health care visit in 2022. We compared differences in patient characteristics associated with having any telemedicine visits vs having an in-person visit only. Then, among telemedicine patients, we identified characteristics associated with having video visits compared with only audio visits. Finally, we examined whether the reasons for and experiences of using telemedicine differed by telemedicine mode.

## Methods

The Advocate Aurora Health institutional review board determined that study was not human participant research, and thus informed consent was not required. This cross-sectional study followed the Strengthening the Reporting of Observational Studies in Epidemiology (STROBE) reporting guideline.^21^

### Data and Study Population

This cross-sectional study used data from the 2022 Health Information National Trends Survey (HINTS), a nationally representative self-report survey of noninstitutionalized, civilian adults administered by the National Cancer Institute. The survey is administered multimodally, using both paper and web-based surveys. Households that were potentially Spanish speaking received materials in English and Spanish while other households were able to request a Spanish survey. HINTS uses survey weights to obtain a nationally representative sample of US adults and to account for nonresponse bias. Data were collected between March 7 and November 8, 2022. The overall response rate was 28.1%.^22^ This study was restricted to adults who had a health care visit in the past 12 months (5437 of 6252 individuals, weighted 86% of adults) to focus on experiences using the health care system.

### Dependent Variables

We measured any telemedicine use (vs only in-person visits) using the question “In the past 12 months, did you receive care from a doctor or health professional using telehealth?” The survey defined telehealth as a “a telephone or video appointment with a doctor or health professional”; it is often referred to as telemedicine. We categorized respondents who answered yes by video, phone call, or both as having had any telemedicine visits, while others were categorized as having in-person visits only. Among individuals who used telemedicine, we categorized those who responded yes by video (or by both video and phone) as having video visits and those who responded yes by phone as having audio-only visits. Those who had telemedicine visits may have also had in-person visits; this was not captured by the survey.

Individuals who used telemedicine were asked 3 questions capturing reasons for using telemedicine and experiences. First, they were asked why they chose a telemedicine visit (yes or no to each): clinician recommendation or requirement, advice on needing in-person care, to avoid infection, convenience, and/or to include others in the appointment. Second, they were asked the primary reason for their most recent telemedicine visit: annual visit; minor illness or acute care; chronic disease management; medical emergency; mental health, behavioral, or substance abuse issues; or other. Finally, they were asked whether they experienced technical problems, whether telemedicine care received was as good as an in-person visit, and if they had privacy concerns regarding their visit. These questions used a 4-point Likert response scale, which we dichotomized based on agreement and disagreement with the statement. Full questions and response options are available in eTable 1 in Supplement 1.

### Independent Variables

Patient characteristics included age, sex, race and ethnicity (Hispanic, non-Hispanic Asian, non-Hispanic Black, non-Hispanic White, or non-Hispanic other [including American Indian or Alaska Native, Other Pacific Islander, or multiple races]), education, marital status, household income, insurance, health status, number of chronic conditions, number of health care visits, and internet use. Living in a linguistically isolated area, urbanicity, and census region were also included.

### Statistical Analysis

We used descriptive statistics and χ^2^ tests to understand differences in characteristics by telemedicine use and telemedicine mode. We also used χ^2^ tests to test differences in reasons for using telemedicine and experiences by telemedicine mode. We used the Bonferroni correction to adjust for multiple comparisons and calculated standardized Pearson residuals where needed.

We conducted 2 analyses using multivariable logistic regression models. First, we assessed differences in characteristics of telemedicine and patients who used only in-person visits. Second, we focused on telemedicine patients and compared characteristics of those who only had an audio-only visit with those who had a video or both types of telemedicine visits. Model goodness-of-fit was confirmed with the area under the receiver operating characteristic curve (which determines the model’s ability to discriminate success and failure), the link test (which determines that the model is properly specified if the prediction squared has no explanatory power),^23^ and the Archer-Lemeshow test (the Hosmer-Lemeshow test adapted for complex survey samples; whether the fitted model describes the observed data).^24^ Weighted missing percentage for sex (5.6%), race or ethnicity (8.2%), education (5.5%), marital status (5.7%), and household income (9.6%) were greater than 5% so we ran models with and without missing as a category. Findings were similar so models without missing categories are presented.

Analyses were conducted using Stata version 17.0 (Stata Corporation). Survey weighting procedures with jackknife replicate weights accounted for the complex survey design; all reported estimates were weighted to represent the US population. Two-sided *P* < .05 was considered statistically significant. Data were analyzed from May to September 2023.

## Results

The sample of 5437 respondents with a health care visit represented more than 216 million adults nationally. In the final weighted sample, 3136 (53%) identified as female and 1928 (47%) identified as male, with a mean (SE) age of 49.4 (0.23) years.

### Telemedicine Visits in the US

We found that 2384 patients (43%) reported that 1 or more of their visits in the past 12 months was through telemedicine. A greater percentage of those with any telemedicine visits were female, had poorer health, had more chronic conditions, used the internet, and had more than 5 health care visits (Table 1). Telemedicine use was reported by 50% of patients with multiple chronic conditions (33% with no chronic conditions) and 45% of patients with internet access (31% without internet) (eTable 2 in Supplement 1). Telemedicine use was reported more often by patients with multiple visits (2-4 visits: 42%; ≥5 visits: 58%) than those with only 1 visit (28%).

**Table 1.** Sociodemographic, Clinical, and Technology Characteristics of Adults With a Health Care Visit in the Past 12 Months, 2022^a^

Characteristic	Patients, No. (weighted %)						
	All (N = 5437)	In-person visit only	Any telemedicine visit	Adjusted *P* value^b^	Any video visit	Audio-only visit	Adjusted *P* value^c^
Total, No. (%)	5437	2933 (57)	2384 (43)	NA	1565 (70)	819 (30)	NA
Age, y							
18-34	746 (23.2)	395 (24.1)	351 (22.0)	.14	256 (23.6)	95 (18.2)	<.001
35-49	1024 (25.0)	493 (21.8)	531 (29.1)		383 (30.8)	148 (25.2)	
50-64	1535 (28.5)	852 (28.9)	683 (27.9)		467 (28.8)	216 (25.7)	
65-74	1195 (14.0)	697 (15.0)	498 (12.7)		307 (11.0)	191 (16.8)	
≥75	756 (9.3)	464 (10.2)	292 (8.3)		138 (5.8)	154 (14.0)	
Sex							
Female	3136 (53.4)	1663 (49.6)	1473 (58.3)	.03	974 (58.1)	499 (58.7)	>.99
Male	1928 (46.6)	1144 (50.4)	784 (41.7)		510 (41.9)	274 (41.3)	
Race or ethnicity							
Non-Hispanic Asian	230 (5.1)	133 (5.0)	97 (5.2)	>.99	60 (5.5)	37 (4.6)	>.99
Non-Hispanic Black	774 (10.9)	448 (11.7)	326 (9.7)		211 (9.8)	115 (9.7)	
Hispanic	806 (15.0)	398 (13.6)	408 (16.9)		246 (15.9)	162 (19.4)	
Non-Hispanic White	2884 (64.8)	1625 (66.0)	1259 (63.3)		868 (63.9)	391 (61.9)	
Non-Hispanic other^d^	154 (4.2)	73 (3.7)	81 (4.8)		55 (4.9)	26 (4.5)	
Education							
College graduate or more	2440 (34.4)	1252 (31.7)	1188 (38.0)	.56	833 (40.2)	355 (32.8)	.42
Some college or vocational	1450 (39.0)	822 (39.6)	628 (38.3)		406 (39.1)	222 (36.4)	
High school graduate	888 (20.9)	559 (22.2)	329 (19.1)		192 (16.9)	137 (24.3)	
Less than high school	291 (5.7)	181 (6.5)	110 (4.6)		50 (3.8)	60 (6.5)	
Marital status							
Married	2318 (53.1)	1266 (51.9)	1052 (54.7)	>.99	714 (56.1)	338 (51.6)	>.99
Not married	2737 (46.9)	1537 (48.1)	1200 (45.3)		761 (43.9)	439 (48.4)	
Household income, $							
≥$75 000	1964 (46.6)	1017 (44.1)	947 (49.8)	>.99	687 (53.1)	260 (42.1)	.08
$35 000 to <$75 000	1443 (29.7)	822 (31.2)	621 (27.7)		399 (27.3)	222 (28.7)	
<$35 000	1388 (23.7)	808 (24.7)	580 (22.5)		331 (19.6)	249 (29.2)	
Insurance							
Covered	4956 (92.7)	2711 (91.3)	2245 (94.4)	.28	1484 (95.9)	761 (91.0)	.01
Not covered	336 (7.3)	207 (8.7)	129 (5.6)		74 (4.1)	55 (9.0)	
Health status							
Excellent, very good, or good	4275 (84.1)	2423 (87.0)	1852 (80.3)	<.001	1234 (81.9)	618 (76.5)	>.99
Poor or fair	941 (15.9)	459 (13.0)	482 (19.7)		301 (18.1)	181 (23.5)	
Chronic conditions^e^							
0	1626 (36.7)	1038 (43.6)	588 (27.7)	<.001	377 (28.0)	211 (26.8)	>.99
≥1	3602 (63.3)	1847 (56.4)	1755 (72.3)		1161 (72.0)	594 (73.2)	
Census region							
Northeast	794 (18.1)	423 (16.9)	371 (19.6)	<.001	260 (19.8)	111 (19.3)	>.99
Midwest	915 (21.0)	595 (24.9)	320 (15.9)		212 (16.7)	108 (14.0)	
South	2418 (38.3)	1385 (39.2)	1033 (37.2)		693 (37.7)	340 (36.1)	
West	1190 (22.6)	530 (19.0)	660 (27.3)		400 (25.8)	260 (30.6)	
Metropolitan status							
Metropolitan	4616 (87.4)	2480 (86.5)	2136 (88.7)	>.99	1407 (88.5)	729 (89.2)	>.99
Nonmetropolitan	701 (12.6)	453 (13.5)	248 (11.3)		158 (11.5)	90 (10.8)	
Living in linguistically isolated strata^f^							
No	4854 (94.6)	2693 (95.1)	2161 (94.0)	>.99	1429 (94.7)	732 (92.3)	.42
Yes	463 (5.4)	240 (4.9)	223 (6.0)		136 (5.3)	87 (7.7)	
Uses internet							
Yes	4494 (87.7)	2393 (85.0)	2101 (91.1)	<.001	1442 (95.0)	659 (82.4)	<.001
No	821 (12.3)	538 (15.0)	283 (8.9)		123 (5.0)	160 (17.6)	
No. of health care visits							
1	850 (18.3)	604 (23.2)	246 (11.8)	<.001	135 (10.8)	111 (14.3)	>.99
2-4	2947 (56.2)	1681 (58.0)	1266 (53.8)		823 (52.7)	443 (56.2)	
≥5	1520 (25.6)	648 (18.8)	872 (34.4)		607 (36.5)	265 (29.5)	

^a^ Data source: Health Information National Trends Survey, 2022. Columns may not sum to group totals due to missing values. Values may be missing because respondents did not answer the question or made an error in their answer (eg, multiple responses selected).

^b^ Bonferroni-adjusted *P* values from χ^2^-tests to test the difference in each characteristic by any telemedicine visit.

^c^ Bonferroni-adjusted *P* values from χ^2^-tests to test the difference in each characteristic by telemedicine mode.

^d^ Non-Hispanic Other includes American Indian or Alaska Native, Other Pacific Islander, or multiple races.

^e^ Chronic conditions included diabetes, high blood pressure, heart condition, lung disease, and depression.

^f^ Lives in a Census tract in which at least 13% of the households are classified as linguistically isolated Spanish-speaking households.

In multivariable analysis (Table 2), having chronic conditions (adjusted odds ratio [aOR], 2.13; 95% CI, 1.66-2.73; *P* < .001), multiple health care visits (2-4: aOR, 1.77; 95% CI, 1.23-2.54; *P* = .003; ≥5: aOR, 3.29; 95% CI, 2.20-4.92; *P* < .001), and female sex (aOR, 1.43; 95% CI, 1.12-1.83; *P* = .006) significantly increased the odds of having any telemedicine visits. Conversely, being in the oldest age group (75 years and older: aOR, 0.63; 95% CI, 0.42-0.94; *P* = .03), having no internet use (aOR, 0.62; 95% CI, 0.48-0.81; *P* < .001), and living in the Midwest (aOR, 0.50; 95% CI, 0.35-0.70; *P* < .001) significantly lowered the odds. We did not find significant differences based on education, race and ethnicity, income, urbanicity, and living in a linguistically isolated area.

**Table 2.** Multivariable Logistic Associations With Any Telemedicine Visit and Audio-Only Telemedicine Visits Among Adults With a Health Care Visit in the Past 12 Months, 2022^a^

Characteristics	Any telemedicine^b^		Audio-only visit^c^	
	AOR (95% CI)	*P* value	AOR (95% CI)	*P* value
Age, y				
18-34	1.00 [Reference]	NA	1.00 [Reference]	NA
35-49	1.42 (0.97-2.08)	.07	1.27 (0.66-2.43)	.47
50-64	0.91 (0.64-1.30)	.61	1.40 (0.74-2.65)	.30
65-74	0.72 (0.47-1.11)	.14	2.13 (1.09-4.14)	.03
≥75	0.63 (0.42-0.94)	.03	3.58 (1.60-8.00)	.002
Sex				
Male	1.00 [Reference]	NA	1.00 [Reference]	NA
Female	1.43 (1.12-1.83)	.006	0.99 (0.67-1.45)	.94
Race or ethnicity				
Asian, non-Hispanic	0.95 (0.45-2.01)	.89	0.90 (0.32-2.52)	.83
Black, non-Hispanic	0.85 (0.61-1.18)	.32	0.98 (0.63-1.53)	.92
Hispanic	1.36 (0.94-1.96)	.10	0.93 (0.56-1.55)	.79
White, non-Hispanic	1.00 [Reference]	NA	1.00 [Reference]	NA
Other race, non-Hispanic^d^	1.19 (0.66-2.15)	.56	0.94 (0.43-2.04)	.87
Education				
College graduate or more	1.00 [Reference]	NA	1.00 [Reference]	NA
Some college or vocational	0.82 (0.61-1.12)	.21	0.98 (0.62-1.55)	.93
High school grad	0.78 (0.57-1.07)	.12	1.39 (0.74-2.61)	.30
Less than high school	0.60 (0.29-1.23)	.16	1.06 (0.50-2.23)	.87
Marital status				
Married	1.00 [Reference]	NA	1.00 [Reference]	NA
Not married	0.87 (0.73-1.05)	.14	1.06 (0.76-1.48)	.73
Household income, $				
≥$75 000	1.00 [Reference]	NA	1.00 [Reference]	NA
$35 000 to <$75 000	0.89 (0.66-1.19)	.42	1.14 (0.72-1.80)	.57
<$35 000	0.89 (0.63-1.24)	.48	1.54 (0.91-2.61)	.11
Insurance				
Covered	1.00 [Reference]	NA	1.00 [Reference]	NA
Not covered	0.69 (0.38-1.24)	.21	2.84 (1.42-5.67)	.004
Health status				
Excellent, very good, or good	1.00 [Reference]	NA	1.00 [Reference]	NA
Poor or fair	1.27 (1.00-1.61)	.05	1.15 (0.73-1.83)	.54
Chronic conditions^e^				
None	1.00 [Reference]	NA	1.00 [Reference]	NA
≥1	2.13 (1.66-2.73)	<.001	0.85 (0.61-1.19)	.35
No. of health care visits	1.00 [Reference]	NA	1.00 [Reference]	NA
1				
2-4	1.77 (1.23-2.54)	.003	0.72 (0.34-1.55)	.40
≥5	3.29 (2.20-4.92)	<.001	0.52 (0.23-1.15)	.11
Internet use				
Yes	1.00 [Reference]	NA	1.00 [Reference]	NA
No	0.62 (0.48-0.81)	<.001	2.11 (1.18-3.78)	.01
Living in high linguistically isolated strata^f^				
Yes	1.20 (0.80-1.79)	.37	1.21 (0.76-1.93)	.41
No	1.00 [Reference]	NA	1.00 [Reference]	NA
Metropolitan status				
Metropolitan	1.00 [Reference]	NA	1.00 [Reference]	NA
Nonmetropolitan	0.92 (0.67-1.26)	.58	0.89 (0.56-1.40)	.61
Census region				
Northeast	1.00 [Reference]	NA	1.00 [Reference]	NA
Midwest	0.50 (0.35-0.70)	<.001	0.86 (0.48-1.53)	.60
South	0.80 (0.59-1.09)	.16	0.94 (0.56-1.59)	.81
West	1.16 (0.81-1.68)	.41	1.23 (0.78-1.95)	.36
Goodness-of-fit				
Area under the curve	0.698	NA	0.672	NA
Link test	NA	.38	NA	.05
Archer-Lemeshow test	2.10^g^	.05	1.77^g^	.11

Abbreviations: AOR, adjusted odds ratio; NA, not applicable.

^a^ Data source: Health Information National Trends Survey, 2022.

^b^ Reference was in-person.

^c^ Reference was video visit.

^d^ Non-Hispanic other includes American Indian or Alaska Native, Other Pacific Islander, or multiple races.

^e^ Chronic conditions included diabetes, high blood pressure, heart condition, lung disease, and depression.

^f^ Lives in a census tract in which at least 13% of the households are classified as linguistically isolated Spanish-speaking households.

^g^ *F*_9,41_.

### Telemedicine Visit Modality (Video vs Audio-Only)

Among the 2384 individuals who used telemedicine, 1565 (70%) had a video visit while 819 (30%) had an audio-only visit. A greater percentage of patients with audio-only visits were older, were uninsured, and did not use the internet (Table 1). While approximately 40% of both the youngest (351 [41%]) and oldest patients (292 [38%]) reported telemedicine use, only 138 patients (49%) of individuals who used telemedicine aged 75 years and older had a video visit compared with 256 (75%) of individuals who used telemedicine aged 18 to 34 years (eTable 2 in Supplement 1).

Multivariable analysis of audio-only vs any video use among individuals who used telemedicine found being in the oldest age groups (aged 65 to 74 years: aOR, 2.13; 95% CI, 1.09-4.14; *P* = .03; ≥75 years: aOR, 3.58; 95% CI, 1.60-8.00; *P* = .002), being uninsured (aOR, 2.84; 95% CI, 1.42-5.67; *P* = .004), and no internet use (aOR, 2.11; 95% CI, 1.18-3.78; *P* = .01) were significantly associated with greater odds of just audio-only telemedicine use (Table 2). Similar to the telemedicine use model results, in the modality model, we observed no significant differences by race and ethnicity, education, marital status, income, health status, living in a linguistically isolated area, and urbanicity. Telemedicine mode also did not significantly differ by number of chronic conditions, number of health care visits, or census region.

### Reasons for and Experiences Using Telemedicine

**Figure 1.**
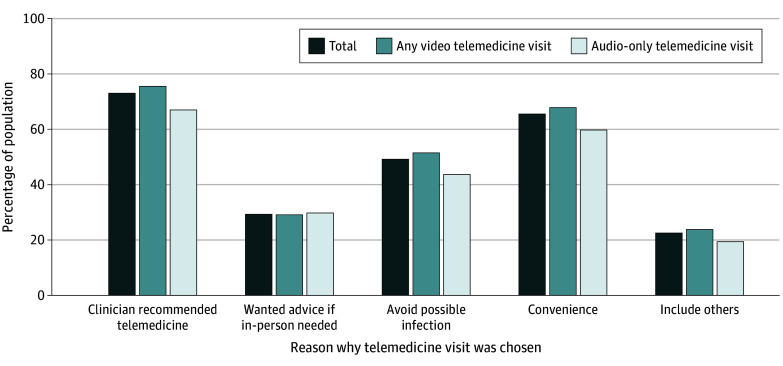
Reasons Why US Adults Chose a Telemedicine Visit, by Telemedicine Mode, Health Information National Trends Survey, 2022 (n = 2384) χ^2^-Tests were used to test the difference in each reason why telemedicine visits were chosen by telemedicine mode.

Individuals who used video and audio-only telemedicine offered similar reasons for choosing a telemedicine visit (Figure 1 and eTable 3 in Supplement 1). The majority of both modes chose telemedicine based on clinician recommendation or requirement (video, 1122 [76%]; audio-only, 497 [67%]) or for convenience (video, 1003 [68%]; audio-only, 424 [60%]). Approximately half wanted to avoid possible infections (1007 [49%]) and almost a third wanted to ask their clinician whether an in-person visit was needed (630 [29%]). More than one-fifth chose telemedicine to include others (446 [22%]).

**Figure 2.**
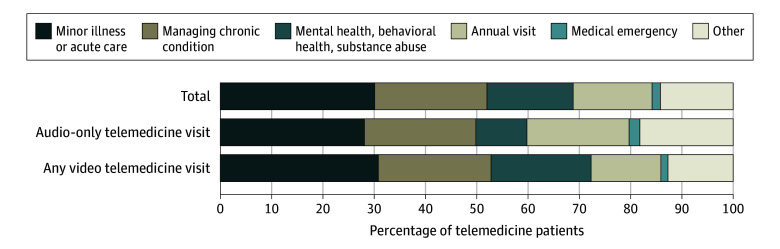
Primary Reasons for Most Recent Telemedicine Visit, by Telemedicine Mode, Health Information National Trends Survey, 2022 The *P* value from the χ^2^-test assessing the difference in primary reason for most recent telemedicine visit by telemedicine mode was *P* = .008. There were statistically significant differences between the observed and expected frequencies based on adjusted residuals for all audio-only and any video telemedicine visit values.

The goal of the most recent visit was often similar across modes (Figure 2 and eTable 3 in Supplement 1). Most often, patients using telemedicine sought acute care (568 [30%]) or chronic condition management (557 [22%]). Patients who only used audio were more likely than patients using video to have used telemedicine for an annual visit (167 [20%] vs 256 [14%]), while patients using video were more likely than patients who only used audio to have used it for a behavioral health visit (270 [20%] vs 55 [10%]). Only 43 patients (2%) used their recent telemedicine visit for medical emergencies. 

**Figure 3.**
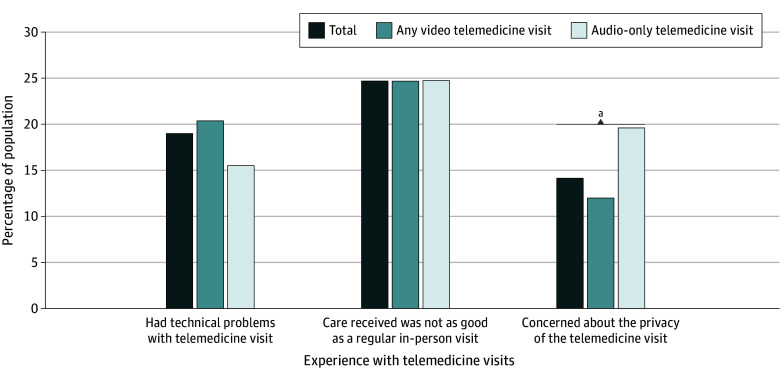
Experience With Telemedicine Visit(s) Among US Adults, by Telemedicine Mode, Health Information National Trends Survey, 2022 (n = 2384) Supporting data are available in eTable 3 in Supplement 1. χ^2^ Tests were used to test the difference in each experience with telemedicine visit by telemedicine mode. ^a^Statistically significant difference (Bonferroni adjusted *P* <.05) by telemedicine mode.

When asked about their experience using telemedicine, 25% of both individuals who used video (375) and those who used only audio (188) thought that their telemedicine care was not as good as in-person care, while 461 (19%) reported experiencing technical problems (Figure 3 and eTable 3 in Supplement 1). Patients who only used audio were more likely to be concerned about the privacy of their telemedicine visit than those who used video (126 [20%] vs 200 [12%]; *P* = .02).

## Discussion

Telemedicine use remained high in 2022, 2 years after the onset of the COVID-19 pandemic. Our findings showed that the use of telemedicine has persisted beyond the period of COVID-19–related restrictions and many patients chose to use telemedicine even when in-person visits were available. Nationally, more than 40% of adults with a health care visit reported a telemedicine visit. Notably, this estimate of telemedicine use is higher than previously reported, likely because previous estimates included all US adults (37% in 2021, 39% in 2022)^5,25^ or used a different recall period (4 weeks instead of the 12 months reported here).^4^

Similar to previous studies,^5,12,13,14,18,20,26^ we found having chronic conditions, multiple health care visits, and female sex increased the odds of having any telemedicine visits while older age, no internet use, and living in the Midwest decreased the odds. The findings associated with age and internet use were consistent with concerns associated with a digital divide, positing that younger, wealthier patients were more likely to adopt digital technologies.^27^ Conversely, patients with more health needs and/or health care interactions were more likely to use telemedicine. Since the management of numerous chronic conditions using telemedicine has been found to be equivalent to in-person care,^28^ health care systems may consider adopting and promoting telemedicine to help clinicians monitor and manage chronic diseases.^29^

Notably, 30% of individuals using telemedicine only used audio, suggesting a substantial desire or need for audio-only access. The multivariate analysis examining characteristics associated with telemedicine mode found patients who were uninsured, older, and did not use the internet being more likely to use only audio telemedicine. In addition to also being consistent with the digital divide concerns, greater use of audio-only visits among uninsured patients may be occurring at safety-net organizations like community health centers.^19,30^ Studies looking at telemedicine use in safety-net organizations have identified both organizational-level barriers (eg, limited IT support, lower operating margins) and patient-level barriers (eg, limited data plans) resulting in greater availability and use of audio-only telemedicine in these settings.^31,32^

Unlike previous studies examining telemedicine use and mode,^4,12,13,16,17^ we did not observe differences by race and ethnicity, education, or income. Our findings were consistent with those associated with use of other health technology use. A 2017 study found no differences in portal use by race and ethnicity or in technology barriers (ie, internet use) by socioeconomic status.^33^ Like portal use, barriers to video telemedicine may be more likely to be associated with individual differences in knowledge, skills, and comfort using technology rather than technology access.^33^ Our findings likely also reflect widespread telemedicine availability resulting from health systems investments in infrastructure and technical support during the COVID-19 pandemic. Future studies will need to continue monitoring these trends to determine if these findings endure.

While other studies have observed greater patient satisfaction among individuals who used video compared with individuals who only used audio,^34,35^ our findings suggested that the reasons for using telemedicine and user experiences were generally similar across modes. The sole exception was that individuals who used only audio were more likely to have privacy concerns about their telemedicine visit. Audio-only calls may provide patients a greater sense of personal privacy (eg, clinician cannot see patients’ homes) and alleviate concerns with digital data privacy.^31,36,37^ To support continued use of audio-only telemedicine, clinicians and health care systems may consider developing system-level policies that prioritize patient privacy and security, such as ensuring support of existing Health Insurance Portability and Accountability Act regulations across all telehealth platforms and providing recommendations for maintaining personal privacy (eg, headphones).^36,37^

While the primary reasons for telemedicine visits in both modalities were minor illnesses and chronic disease management, other reasons differed by modality. Like previous studies, we observed a greater percentage of individuals who used only audio reporting an annual visit, potentially because these visits were often provided by clinicians with whom patients have an established relationship.^38^ We observed a greater proportion of individuals who used video with a behavioral health visit. The shift to video telemedicine for behavioral health care may reflect the confluence of greater patient willingness to use and comfort with video telemedicine and clinician preference for video care.^9,31^ Video behavioral health visits offer clinicians much of the same visual information provided through in-person visits, such as nonverbal cues, though video visits have the added advantage of allowing clinicians to view patients’ home environments to tailor their treatments.^31,39^

Notably, only 25% of both individuals who used video and audio-only telemedicine felt that the care they received via telemedicine was not as good as in-person care. Similarly, Steelfisher et al^9^ found that only 33% of individuals who used video telemedicine perceived that the quality of their care was worse compared with in-person visits, largely because of the clinician’s inability to conduct a physical examination. The authors also found that although 90% of clinicians felt their video visits went well, 80% of clinicians would prefer to provide little or no care via telemedicine; 60% of clinicians felt that the quality of care provided by video was worse than in-person care.^9^ These findings suggest that clinician preferences to move away from telemedicine may be the largest barrier to receiving care via telemedicine. Future studies will need to better understand how health care system and clinician preferences and concerns contribute to telemedicine use among patients and how visit modality affects care quality and health outcomes. At least in the short run, health care systems may want to focus their efforts on promoting the use of telemedicine for select services, such as chronic condition updates, medication refills, and behavioral health visits that do not require physical examinations.^7,9^

While video telemedicine is likely to persist, the future of audio-only visits is less certain. The Centers for Medicaid & Medicare Services (CMS) has permanently extended reimbursement for both video and audio-only behavioral health telemedicine visits for Medicare patients, but reimbursement for other telemedicine visits were expected to end after December 2024.^40^ Furthermore, reimbursement for audio-only visits were limited to a smaller set of services, including evaluation and management, behavioral health, and education services.^40,41^ Telemedicine policies affecting patients with Medicaid or commercial insurance coverage vary by state and insurer.^42^ Many state and commercial insurers have already started limiting the scope and reach of audio-only telemedicine services.^43,44^ Given the characteristics of the patients who were more likely to use audio-only services, these restrictions may disproportionately hinder remote access among disadvantaged patient populations (ie, the poor elderly, those lacking access or ability to use the internet, the uninsured). Policies to improve broadband access, including the Emergency Broadband Benefit Program and the Infrastructure Investment and Jobs Act provide $3.2 billion and $65 billion, respectively, will help to improve access to broadband and internet-enabled devices, thus enabling more equitable access to video visits.^42^ At the health care system–level, patients could likely benefit from direct assistance in using new technologies, including access to internet-enabled devices and education on using digital health tools effectively.^10^

### Limitations

This study has limitations. First, the survey does not allow us to determine whether not using telemedicine is due to availability or patient preference. Second, self-reported survey responses may be subject to recall bias and because the design is cross-sectional, it does not allow us to make causal claims. Third, while the low response rate may raise concerns associated with nonrespondent bias, the sampling and weighting strategy used by HINTS is intended to minimize biases and improve national representativeness and generalizability of the findings. Finally, because of HINTS survey limitations, we could not ascertain whether patients with telemedicine visits also had in-person visits or assess patients’ satisfaction with their in-person visit experiences.

## Conclusions

Our study found that 2 years after the onset of the COVID-19 pandemic, telemedicine was still used by over two-fifths of adult patients nationally, notably among patients with greater care needs, suggesting that telemedicine could endure as a satisfactory option in health care delivery. We did not observe differences in telemedicine use or mode by several common measures of socioeconomic status (eg, race and ethnicity, education, income), which may reflect the widespread use of electronic devices and successful national and health care system efforts to maximize telemedicine adoption. However, access to telemedicine, and video visits more specifically, is still less likely among key portions of the population who experience other logistical barriers to accessing care. Continued monitoring of technology improvements, who is using telemedicine, and for what care needs, is needed to support policy makers and health care systems advancing policies and systems that promote access to health care innovations.

## Notes

Supplement 1. **eTable 1.** Health Information National Trends Survey 6 Questions, 2022 **eTable 2.** Telemedicine Visits and Mode by Patient Characteristics Among Adults With a Health Care Visit in the Past 12 Months, 2022 **eTable 3.** Reasons For and Experiences Using Telemedicine by Telemedicine Mode, Among Adults With a Health Care Visit and a Telemedicine Visit in the Past 12 Months, 2022

Supplement 2.
Data Sharing Statement

## References

[1] Harju A, Neufeld J. Telehealth utilization during the COVID-19 pandemic: a preliminary selective review. Telemed Rep. 2022;3(1):38-47. doi:10.1089/tmr.2021.0040 35720447 PMC8989093

[2] Patel SY, Mehrotra A, Huskamp HA, Uscher-Pines L, Ganguli I, Barnett ML. Trends in outpatient care delivery and telemedicine during the COVID-19 pandemic in the US. JAMA Intern Med. 2021;181(3):388-391. doi:10.1001/jamainternmed.2020.5928 33196765 PMC7670397

[3] Mehrotra A, Chernew ME, Linetsky D, Hatch H, Cutler DA, Scheider EC. The impact of COVID-19 on outpatient visits in 2020: Visits remained stable, despite a late surge in cases. Commonwealth Fund. Accessed March 10, 2022. https://www.commonwealthfund.org/publications/2021/feb/impact-covid-19-outpatient-visits-2020-visits-stable-despite-late-surge

[4] Lee EC, Grigorescu V, Smith SR, Samson LW, Conmy AB, De Lew N. Updated National Survey Trends in Telehealth Utilization and Modality: 2021-2022 (Issue Brief No. HP-2023-09). US Department of Health and Human Services. Accessed February 13, 2024. https://aspe.hhs.gov/sites/default/files/documents/7d6b4989431f4c70144f209622975116/household-pulse-survey-telehealth-covid-ib.pdf

[5] Chandrasekaran R. Telemedicine in the post-pandemic period: understanding patterns of use and the influence of socioeconomic demographics, health status, and social determinants. Telemed J E Health. Published online August 19, 2023. doi:10.1089/tmj.2023.0277 37585558

[6] Fischer SH, Predmore Z, Roth E, Uscher-Pines L, Baird M, Breslau J. Use of and willingness to use video telehealth through the COVID-19 pandemic. Health Aff (Millwood). 2022;41(11):1645-1651. doi:10.1377/hlthaff.2022.00118 36343311

[7] Ebbert JO, Ramar P, Tulledge-Scheitel SM, . Patient preferences for telehealth services in a large multispecialty practice. J Telemed Telecare. 2023;29(4):298-303. doi:10.1177/1357633X20980302 33461397

[8] Predmore ZS, Roth E, Breslau J, Fischer SH, Uscher-Pines L. Assessment of patient preferences for telehealth in post-COVID-19 pandemic health care. JAMA Netw Open. 2021;4(12):e2136405. doi:10.1001/jamanetworkopen.2021.36405 34851400 PMC8637257

[9] SteelFisher GK, McMurtry CL, Caporello H, . Video telemedicine experiences in COVID-19 were positive, but physicians and patients prefer in-person care for the future. Health Aff (Millwood). 2023;42(4):575-584. doi:10.1377/hlthaff.2022.01027 37011316 PMC11154740

[10] Nouri S, Khoong EC, Lyles CR, Karliner L. Addressing equity in telemedicine for chronic disease management during the COVID-19 pandemic. NEJM Catalyst. Published online May 4, 2020. doi:10.1056/CAT.20.0123

[11] Patel SY, Mehrotra A, Huskamp HA, Uscher-Pines L, Ganguli I, Barnett ML. Variation in telemedicine use and outpatient care during the COVID-19 pandemic in the United States. Health Aff (Millwood). 2021;40(2):349-358. doi:10.1377/hlthaff.2020.01786 33523745 PMC7967498

[12] Sachs JW, Graven P, Gold JA, Kassakian SZ. Disparities in telephone and video telehealth engagement during the COVID-19 pandemic. JAMIA Open. 2021;4(3):ooab056. doi:10.1093/jamiaopen/ooab056 34632322 PMC8496485

[13] Eberly LA, Kallan MJ, Julien HM, . Patient Characteristics associated with telemedicine access for primary and specialty ambulatory care during the COVID-19 pandemic. JAMA Netw Open. 2020;3(12):e2031640. doi:10.1001/jamanetworkopen.2020.31640 33372974 PMC7772717

[14] Rodriguez JA, Saadi A, Schwamm LH, Bates DW, Samal L. Disparities in telehealth use among California patients with limited English proficiency. Health Aff (Millwood). 2021;40(3):487-495. doi:10.1377/hlthaff.2020.00823 33646862

[15] Reed ME, Huang J, Graetz I, . Patient characteristics associated with choosing a telemedicine visit vs office visit with the same primary care clinicians. JAMA Netw Open. 2020;3(6):e205873. doi:10.1001/jamanetworkopen.2020.5873 32585018 PMC7301227

[16] Rodriguez JA, Betancourt JR, Sequist TD, Ganguli I. Differences in the use of telephone and video telemedicine visits during the COVID-19 pandemic. Am J Manag Care. 2021;27(1):21-26. doi:10.37765/ajmc.2021.88573 33471458 PMC10877492

[17] Chen J, Li KY, Andino J, . Predictors of audio-only versus video telehealth visits during the COVID-19 pandemic. J Gen Intern Med. 2022;37(5):1138-1144. doi:10.1007/s11606-021-07172-y 34791589 PMC8597874

[18] Chang E, Davis TL, Berkman ND. Differences in telemedicine, emergency department, and hospital utilization among nonelderly adults with limited english proficiency post-COVID-19 pandemic: a cross-sectional analysis. J Gen Intern Med. 2023;38(16):3490-3498. doi:10.1007/s11606-023-08353-7 37592119 PMC10713935

[19] Chang JE, Lai AY, Gupta A, Nguyen AM, Berry CA, Shelley DR. Rapid transition to telehealth and the digital divide: implications for primary care access and equity in a post-COVID era. Milbank Q. 2021;99(2):340-368. doi:10.1111/1468-0009.12509 34075622 PMC8209855

[20] Hossain M, Dean EB, Kaliski D. Using administrative data to examine telemedicine usage among Medicaid beneficiaries during the coronavirus disease 2019 pandemic. Med Care. 2022;60(7):488-495. doi:10.1097/MLR.0000000000001723 35679172 PMC9172580

[21] von Elm E, Altman DG, Egger M, Pocock SJ, Gøtzsche PC, Vandenbroucke JP; STROBE Initiative. The Strengthening the Reporting of Observational Studies in Epidemiology (STROBE) statement: guidelines for reporting observational studies. Ann Intern Med. 2007;147(8):573-577. doi:10.7326/0003-4819-147-8-200710160-00010 17938396

[22] Health Information National Trends Survey 6 (HINTS 6), 2022: public-use data file and documentation. National Cancer Institute. Accessed February 13, 2024. https://hints.cancer.gov/

[23] StataCorp. Stata 17 Base Reference Manual. Stata Press; 2021. Accessed February 19, 2024. https://www.stata.com/manuals/r.pdf

[24] Archer KJ, Lemeshow S. Goodness-of-fit test for a logistic regression model fitted using survey sample data. Stata J. 2006;6(1):97-105. doi:10.1177/1536867X0600600106

[25] Lucas J, Villarroel M. Telemedicine use among adults: United States, 2021. NCHS Data Brief, no 445. National Center for Health Statistics; 2022. 36255940

[26] Friedman AB, Gervasi S, Song H, . Telemedicine catches on: changes in the utilization of telemedicine services during the COVID-19 pandemic. Am J Manag Care. 2022;28(1):e1-e6. doi:10.37765/ajmc.2022.88771 35049260

[27] Perrin A, Atske S. 7% of Americans don’t use the internet: who are they? Pew Research Center. Accessed September 27, 2023. https://www.pewresearch.org/short-reads/2021/04/02/7-of-americans-dont-use-the-internet-who-are-they/

[28] Hanlon P, Daines L, Campbell C, McKinstry B, Weller D, Pinnock H. Telehealth interventions to support self-management of long-term conditions: a systematic metareview of diabetes, heart failure, asthma, chronic obstructive pulmonary disease, and cancer. J Med Internet Res. 2017;19(5):e172. doi:10.2196/jmir.6688 28526671 PMC5451641

[29] Corbett JA, Opladen JM, Bisognano JD. Telemedicine can revolutionize the treatment of chronic disease. Int J Cardiol Hypertens. 2020;7:100051. doi:10.1016/j.ijchy.2020.100051 33330846 PMC7490579

[30] Uscher-Pines L, Arora N, Jones M, . Experiences of Health Centers in Implementing Telehealth Visits for Underserved Patients During the COVID-19 Pandemic: Results from the Connected Care Accelerator Initiative. RAND Corporation; 2022. 10.7249/rhq-09-04-02 PMC9519102 36238021

[31] Chang JE, Lindenfeld Z, Albert SL, . Telephone vs. video visits during COVID-19: safety-net provider perspectives. J Am Board Fam Med. 2021;34(6):1103-1114. doi:10.3122/jabfm.2021.06.210186 34772766

[32] Nguyen OT, Watson AK, Motwani K, . Patient-Level factors associated with utilization of telemedicine services from a free clinic during COVID-19. Telemed J E Health. 2021;28(4):526-534. doi:10.1089/tmj.2021.0102 34255572 PMC13215056

[33] Anthony DL, Campos-Castillo C, Lim PS. Who isn’t using patient portals and why: evidence and implications from a national sample of US adults. Health Aff (Millwood). 2018;37(12):1948-1954. doi:10.1377/hlthaff.2018.05117 30633673

[34] Chen K, Lodaria K, Jackson HB. Patient satisfaction with telehealth versus in-person visits during COVID-19 at a large, public healthcare system. J Eval Clin Pract. 2022;28(6):986-990. doi:10.1111/jep.13770 36148479 PMC9538919

[35] Hays RD, Skootsky SA. Patient experience with in-person and telehealth visits before and during the COVID-19 pandemic at a large integrated health system in the United States. J Gen Intern Med. 2022;37(4):847-852. doi:10.1007/s11606-021-07196-4 34982370 PMC8725638

[36] Ortega G, Rodriguez JA, Maurer LR, . Telemedicine, COVID-19, and disparities: Policy implications. Health Policy Technol. 2020;9(3):368-371. doi:10.1016/j.hlpt.2020.08.001 32837888 PMC7428456

[37] Wood BR, Young JD, Abdel-Massih RC, . Advancing digital health equity: a policy paper of the infectious diseases society of america and the HIV medicine association. Clin Infect Dis. 2021;72(6):913-919. doi:10.1093/cid/ciaa1525 33033829 PMC7665352

[38] Blavin F, Hill I, Smith LB, O’Brien C. How adults with chronic health conditions experience telehealth: insights from the first year of the COVID-19 pandemic. Accessed February 19, 2024. https://www.urban.org/research/publication/how-adults-chronic-health-conditions-experience-telehealths

[39] Chen PV, Helm A, Caloudas SG, . Evidence of phone vs video-conferencing for mental health treatments: a review of the literature. Curr Psychiatry Rep. 2022;24(10):529-539. doi:10.1007/s11920-022-01359-8 36053400 PMC9437398

[40] Health Resources and Services Administration. Telehealth policy changes after the COVID-19 public health emergency. Department of Health and Human Services. Accessed August 28, 2023. https://telehealth.hhs.gov/providers/telehealth-policy/policy-changes-after-the-covid-19-public-health-emergency

[41] Telehealth for providers: what you need to know. US Department of Health and Human Services. Accessed February 13, 2024. https://www.cms.gov/files/document/telehealth-toolkit-providers.pdf

[42] Fact Sheet HHS. Telehealth flexibilities and resources and the COVID-19. Public Health Emergency. Accessed February 13, 2024. https://www.hhs.gov/about/news/2023/05/10/hhs-fact-sheet-telehealth-flexibilities-resources-covid-19-public-health-emergency.html

[43] State telehealth laws and reimbursement policies: Spring 2023 summary chart of key telehealth policy areas. Center for Connected Health Policy. Accessed August 29, 2023. https://www.cchpca.org/2023/05/Spring2023_SummaryChart.pdf

[44] Large national payer to change audio-only telehealth policy. American Academy of Family Physicians. Accessed August 29, 2023. https://www.aafp.org/pubs/fpm/blogs/gettingpaid/entry/uhc-audio-telehealth.html

