# Supplementary material for: Patient Characteristics and Telemedicine Use in the US, 2022
Source: JAMA Netw Open. 2024 Mar 22;7(3):e243354. doi: 10.1001/jamanetworkopen.2024.3354 (PMC12285594; doi:10.1001/jamanetworkopen.2024.3354)
Supplement: Supplement 1. — eTable 1. Health Information National Trends Survey 6 Questions, 2022 eTable 2. Telemedicine Visits and Mode by Patient Characteristics Among Adults With a Health Care Visit in the Past 12 Months, 2022 eTable 3. Reasons For and Experiences Using Telemedicine by Telemedicine Mode, Among Adults With a Health Care Visit and a Telemedicine Visit in the Past 12 Months, 2022 [file jamanetwopen-e243354-s001.pdf]

Online Supplemental Content

Chang E, Penfold R, Berkman NM. Patient characteristics and telemedicine use in the US, 2022. *JAMA Netw Open*. 2024;7(3):e243354. doi:10.1001/jamanetworkopen.2024.3354

eTable 1. Health Information National Trends Survey 6 Questions, 2022
eTable 2. Telemedicine Visits and Mode by Patient Characteristics Among Adults With a Health Care Visit in the Past 12 Months, 2022
eTable 3. Reasons For and Experiences Using Telemedicine by Telemedicine Mode, Among Adults With a Health Care Visit and a Telemedicine Visit in the Past 12 Months, 2022

This supplemental material has been provided by the authors to give readers additional information about their work.

© 2024 Chang E et al. *JAMA Network Open.*

eTable 1. Health Information National Trends Survey 6 Questions, 2022

Measure	HINTS survey question	Variable definition
Had a healthcare visit	C1. In the past 12 months, not counting times you went to an emergency room, how many times did you go to a doctor, nurse, or other health professional to get care for yourself?<br>(None / 1 time / 2 times / 3 times / 4 times / 5-9 times / 10 or more times)	*Had a healthcare visit* coded as "No" if respondents answered "None" and "Yes" otherwise. Those coded as "No" were not included in the analysis
Any telemedicine use; telemedicine mode	D1. A telehealth visit is a telephone or video appointment with a doctor or health professional. In the past 12 months, did you receive care from a doctor or health professional using telehealth?<br>(Yes, by video / Yes, by phone call (voice only with no video) / Yes, some by video and some by phone call / No telehealth visits in the past 12 months)	*Any telemedicine use* coded as "Yes" if respondents answered "Yes, by video", "Yes, by phone call (voice only with no video)", or "Yes, some by video and some by phone call" and "No, in-person visit only" if the respondents answered "No telehealth visits in the past 12 months".<br>*Telemedicine mode* coded as "Video visits" if respondents answered "Yes, by video" or "Yes, some by video and some by phone call" and "Audio-only visits" if respondents answered "Yes, by phone call (voice only with no video)".
Reasons why chose a telemedicine visit	D4. Why did you choose a telehealth visit(s) for yourself? (Yes / No)<br>a. [provider recommended] The health care provider recommended or required the visit use telehealth.<br>b. [wanted advice if in-person needed] I wanted advice about whether I needed in-person medical care.<br>c. [avoid possible infection] I wanted to avoid possible infection at the doctor's office or hospital (for example, COVID-19 or flu).<br>d. [convenience] It was more convenient than going to the doctor (for example, less travel or wait times).<br>e. [include others] I could include family or other caregivers in my appointment.	Each measure was coded as "Yes" if respondents answered "Yes" and "No" otherwise.

Measure	HINTS survey question	Variable definition
Primary reason for most recent telemedicine visit	D5. What was the primary reason for your most recent telehealth visit? (Mark only one)<br>a. Annual visit<br>b. Minor illness/acute care (for example, fever, sinus infection)<br>c. Managing my chronic health condition/disease (for example, high blood pressure, diabetes, heart disease, obesity, cancer)<br>d. Medical emergency<br>e. Mental health, behavioral, or substance abuse issues (for example, depression, anxiety, drug or alcohol abuse)<br>f. Other	
Telemedicine visit experience	D6. In general, how much do you agree or disagree with the following statements regarding your telehealth visit(s)? (Strongly agree / Somewhat agree / Somewhat disagree / Strongly disagree)<br>a. [had technical problems with telemedicine visit] I had technical problems with my telehealth visit(s) (for example, difficulty using the technology, trouble seeing or hearing my health care provider).<br>b. [care received not as good as a regular in-person visit] The care I received through telehealth was as good as a regular in-person visit.<br>c. [privacy concerns] I was concerned about the privacy of my telehealth visit(s).	Each measure was coded as “Agree” if respondents answered “Strongly agree” or “Somewhat agree” and “Disagree” otherwise.<br>For “care received not as good as a regular in-person visit”, responses were then reverse coded for alignment with the other measures.

eTable 2. Telemedicine Visits and Mode by Patient Characteristics Among Adults With a Health Care Visit in the Past 12 Months, 2022

	Weighted % of patients					
				Telemedicine mode		
Characteristic	In-person visit only (57%, N=2,933)	Any telemedicine visit (43%, N=2,384)	Adjusted p-value[a]	Any video visit (70%, N=1,565)	Audio-only visit (30%, N=819)	Adjusted p-value[b]
Age (years)			0. 14			<0.001
18-34	58.8	41.2		75.1	24.9	
35-49	49.4	50.6		74.0	26.0	
50-64	57.6	42.4		72.3	27.7	
65-74	60.6	39.4		60.4	39.6	
≥75	61.7	38.3		49.0	51.0	
Gender			0.03			1.00
Female	52.8	47.2		69.8	30.2	
Male	61.4	38.6		70.3	29.7	
Race/ethnicity			1.00			1.00
Non-Hispanic White	57.7	42.3		71.3	28.7	
Non-Hispanic Black	61.2	38.8		70.8	29.2	
Hispanic	51.2	48.8		66.4	33.6	
Non-Hispanic Asian	55.5	44.5		74.4	25.6	
Non-Hispanic Other[c]	50.3	49.7		72.6	27.4	
Education			0.56			0.42
College graduate or more	52.5	47.5		74.2	25.8	
Some college/vocational	57.7	42.3		71.6	28.4	
High school graduate	60.6	39.4		61.9	38.1	
Less than high school	64.9	35.1		58.1	41.9	
Marital status			1.00			1.00
Married	55.6	44.4		71.5	28.5	
Not married	58.4	41.6		67.7	32.3	
Household income			1.00			0.08
≥$75K	53.4	46.6		74.8	25.2	
$35K to <$75K	59.3	40.7		69.2	30.8	
<$35K	58.8	41.2		61.2	38.8	
Insurance			0.28			0.01
Covered	55.8	44.2		70.8	29.2	
Not covered	66.9	33.1		51.4	48.6	
Health status			<0.001			1.00
Excellent/very good/good	58.5	41.5		71.3	28.7	
Poor/fair	46.2	53.8		64.1	35.9	
Chronic conditions[d]			<0.001			1.00
0	67.2	32.8		70.7	29.3	
≥1	50.4	49.6		69.4	30.6	

	Weighted % of patients					
				Telemedicine mode		
Characteristic	In-person visit only (57%, N=2,933)	Any telemedicine visit (43%, N=2,384)	Adjusted p-value[a]	Any video visit (70%, N=1,565)	Audio-only visit (30%, N=819)	Adjusted p-value[b]
Number of healthcare visits			<0.001			1.00
1	71.9	28.1		63.5	36.5	
2-4	58.5	41.5		68.4	31.6	
5+	41.7	58.3		74.0	26.0	
Uses internet			<0.001			<0.001
Yes	54.9	45.1		72.6	27.4	
No	68.9	31.1		39.7	60.3	
Census region			<0.001			1.00
Northeast	53.0	47.0		70.3	29.7	
Midwest	67.1	32.9		73.3	26.7	
South	57.9	42.1		70.6	29.4	
West	47.6	52.4		66.0	34.0	
Metropolitan status			1.00			1.00
Metropolitan	56.0	44.0		69.6	30.4	
Nonmetropolitan	61.0	39.0		70.9	29.1	
Living in high linguistically isolated strata[e]			1.00			0.42
No	56.9	43.1		70.3	29.7	
Yes	51.5	48.5		61.5	38.5	

Data Source: Health Information National Trends Survey, 2022

a. Bonferroni-adjusted p-values from χ^2-tests to test the difference in each characteristic by any telemedicine visit.

b. Bonferroni-adjusted p-values from χ^2-tests to test the difference in each characteristic by telemedicine mode.

c. Non-Hispanic Other includes American Indian or Alaska Native, Other Pacific Islander, or multiple races.

d. Chronic conditions included diabetes, high blood pressure, heart condition, lung disease, and depression.

e. Lives in a census tract in which at least 13% of the households are classified as linguistically isolated Spanish-speaking households.

eTable 3. Reasons For and Experiences Using Telemedicine by Telemedicine Mode, Among Adults With a Health Care Visit and a Telemedicine Visit in the Past 12 Months, 2022

	N (weighted percent) of telemedicine patients			
	Total	Any video	Audio-only	P value[a]
Reason why telemedicine visit chosen				
Provider recommend/required telemedicine				0.20
Yes	1619 (73.0)	1122 (75.5)	497 (67.0)	
No	633 (27.0)	390 (24.5)	243 (33.0)	
Wanted advice if in-person needed				1.00
Yes	630 (29.3)	417 (29.1)	213 (29.8)	
No	1614 (70.7)	1093 (70.9)	521 (70.2)	
Avoid possible infection				0.35
Yes	1077 (49.2)	753 (51.4)	324 (43.7)	
No	1179 (50.8)	761 (48.6)	418 (56.3)	
Convenience				0.05
Yes	1427 (65.5)	1003 (67.8)	424 (59.7)	
No	825 (34.5)	508 (32.2)	317 (40.3)	
Include others				1.00
Yes	446 (22.5)	311 (23.8)	135 (19.4)	
No	1800 (77.5)	1196 (76.2)	604 (80.6)	
Primary reason for most recent telemedicine visit				0.008
Annual visit	423 (15.4)	256 (13.6)	167 (19.9)	
Minor illness/acute care	568 (30.1)	383 (30.8)	185 (28.1)	
Managing chronic condition	557 (21.9)	378 (22.0)	179 (21.7)	
Medical emergency	43 (1.6)	27 (1.4)	16 (2.1)	
Mental health, behavioral health, substance abuse	325 (16.8)	270 (19.5)	55 (10.0)	
Other	327 (14.2)	194 (12.7)	133 (18.2)	
Experience with telemedicine visit				
Had technical problems with telemedicine visit				0.18
Yes	461 (19.0)	328 (20.4)	133 (15.5)	
No	1724 (81.0)	1154 (79.6)	570 (84.5)	
Care received was not as good as a regular in-person visit				1.00
Yes	563 (24.7)	375 (24.7)	188 (24.7)	
No	1670 (75.3)	1131 (75.3)	539 (75.3)	
Privacy concerns				0.015
Yes	326 (14.1)	200 (12.0)	126 (19.6)	
No	1852 (85.9)	1279 (88.0)	573 (80.4)	

Data Source: Health Information National Trends Survey, 2022

a. P-values for reason why telemedicine visit chosen and experience with telemedicine visit are Bonferroni-adjusted.
