# Supplementary material for: Patient Characteristics and Telemedicine Use in the US, 2022
Source: JAMA Netw Open. 2024 Mar 22;7(3):e243354. doi: 10.1001/jamanetworkopen.2024.3354 (PMC12285594; doi:10.1001/jamanetworkopen.2024.3354)
Supplement: Supplement 2. — Data Sharing Statement [file jamanetwopen-e243354-s002.pdf]

Data Sharing Statement

Chang. Patient Characteristics and Telemedicine Use in the US, 2022. *JAMA Netw Open*. Published March 22, 2024. doi:10.1001/jamanetworkopen.2024.3354

Data
Data available: No

Additional Information
Explanation for why data not available: The data used for this analysis are publicly available at the National Cancer Institute (https://hints.cancer.gov/).
